# Towards Information Inequalities for Generalized Graph Entropies

**DOI:** 10.1371/journal.pone.0038159

**Published:** 2012-06-08

**Authors:** Lavanya Sivakumar, Matthias Dehmer

**Affiliations:** 1 Institute of Mathematical Sciences, Chennai, India; 2 Institute for Bioinformatics and Translational Research, UMIT, Hall in Tyrol, Austria; Technical University of Madrid, Italy

## Abstract

In this article, we discuss the problem of establishing relations between information measures for network structures. Two types of entropy based measures namely, the Shannon entropy and its generalization, the Rényi entropy have been considered for this study. Our main results involve establishing formal relationships, by means of inequalities, between these two kinds of measures. Further, we also state and prove inequalities connecting the classical partition-based graph entropies and partition-independent entropy measures. In addition, several explicit inequalities are derived for special classes of graphs.

## Introduction

Complexity of a system, in general, deals with the intricate design and complex interrelations among the components of the system. Complexity analysis can be categorized in three types based on functional behavior, topological properties, and/or at the compositional level of a system [1]. Over the years, all these categories have been implemented and contemplated concurrently in several branches of science and social science. In this paper, we study the complexity of graphs with respect to its underlying structure. It is often referred to as topological complexity [2], as the measures are used to associate high complexity with low symmetry and larger diversity of the system’s components, while low complexity is related to high symmetry, uniformity and lack of diversity. The quantitative estimation (using measures/indices) of topological complexity has been proven useful when characterizing the networks and has widely spread into all branches of natural sciences, mathematics, statistics, economics and sociology; for e.g., see [3]–[12].

In the study of complexity, information theory has been playing a predominant role. That is, the measures based on Shannon entropy have been very powerful and useful in determining the structural complexity of networks; see [1], [2], [10], [13]. Apart from Shannon entropy, its generalizations such as Rényi entropy [14], Daròczy entropy [15] have also been identified as useful measures for characterizing network-based systems; see [16].

In this paper, we deal with a novel aspect when analyzing the complexity of network-based systems. Namely, we establish relations between information-theoretic complexity measures [17], [18]. Investigating relations (in the form of inequalities) among measures is useful when studying large scale networks where evaluating the exact value of a measure might be computationally challenging. In addition, they also serve as a tool for solving problems: In the field of communication theory, the study of inequalities has led to the development of so-called algebra of information where several rules have been established between the mutual information among events [19] and their respective entropy measures. For example, Young’s inequality, Brunn-Minkowski inequality, Fisher’s information inequalities to name a few in this context [20]–[22].

Inequalities involving information measures for graphs are also referred to as *information inequalities* [23]. They can be classified in two types, namely *implicit information inequalities* and *explicit information inequalities*. In particular, when information measures are present on either side of the inequality, we call it an *implicit information inequality* [23], while in the latter, the information measure is bounded by a function of parameters (or constants) involved. For some of the recent contributions in this direction, we refer to [17], [23]–[26].

Recently, we have established relations [17] involving only Shannon entropy measures, under certain assumptions. In this article we extend the study to analyze the relation between entropy measures belonging to different concepts. In particular, the main contribution of this paper, is to establish implicit information inequalities involving Shannon entropy and Rényi entropy measures when being applied to networks. Further, we present implicit inequalities between Rényi entropy measures having two different types of probability distributions with additional assumptions. To achieve this, we analyze and establish relations between classical partition-based graph entropies [13], [24], [27] and non-partition-based (or the functional) based entropies [28]. Finally, we apply the obtained inequalities to specific graph classes and derive simple explicit bounds for the Rényi entropy.

## Methods

In this section, we state some of the definitions of information-theoretic complexity measures [18], [29]–[31]. These measures are based on two major classifications, namely partition-based and partition-independent measures. Some basic results on inequalities on real numbers [22], [32] are also presented at the end of the section.

Let 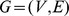 be a graph on *N* vertices where 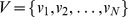 and 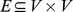. Throughout this article, *G* denotes a simple undirected graph. Let *X* be a collection of subsets of *G* representing a graph object. Let 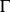 be an equivalence relation that partitions *X* into *k* subsets 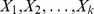, with cardinality 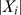, for 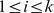. Let 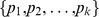 denote the probability distribution on *X* w.r.t 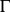, such that 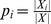 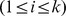, is the value of probability on each of the partition.

For graphs, the Shannon’s entropy measure [33] is also referred to as *the information content of graphs* [13], [27], [34] and is defined as follows:

**Definition 1** The mean information content, 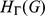, of *G* with respect to 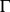 is given by.
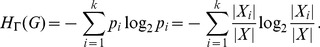
(1)


Note that while the above definition is based on partitioning a graph object, another class of Shannon entropy has been defined in [29] where the probability distribution is independent of partitions. That is, probabilities were defined for every vertex of the graph using the concept of information functionals.

Suppose 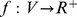 is an arbitrary information functional [29] that maps a set of vertices to the non-negative real numbers and let.
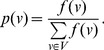
(2)

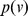
 is the probability value of 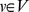.

**Definition 2** *The graph entropy, 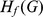, representing the structural information content of G* [*18]*, [*29*] *is then given by,*

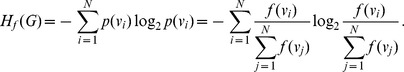
(3)


As a follow-up to Shannon’s seminal work [31], many generalizations of the entropy measure were proposed in the literature [14], [15], [35]. These generalized entropies were recently [16], extended to study graphs. In the following, we present one such generalization from [16], namely the Rényi entropy for graphs.

**Definition 3** *The Rényi entropy 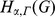, for 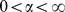 and 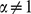, of a graph G* [*16*] *is given by,*

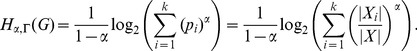
(4)Here, 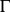 is the equivalence relation on a graph object and 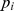 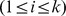 denotes the probabilities defined on the partition induced by 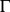.

It has been proved that Rényi entropy is a generalization of Shannon entropy and in the limiting case when 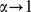, the Rényi entropy equals the Shannon entropy [35].

Similar to expression (3), the Rényi entropy can be immediately extended [16] to partition-independent probability distributions defined on *G*.

**Definition 4** *Let 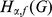, for 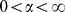 and 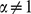, denote the Rényi entropy* [*16*] *defined using an information functional f. Then.*

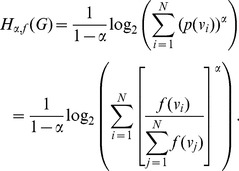
(5)


Next we state some interesting inequalities from the literature that are crucial to prove our main results. One of the well-known result for real numbers is stated as follows [32].

**Lemma 1** [32] *Let* 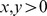 *and* 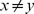 *be real numbers. Then.*

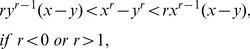
(6)

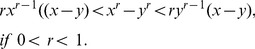
(7)


A simplified form of Minkowski’s inequality has been expressed in [32].

**Lemma 2** [32] *If* 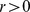 *, then.*

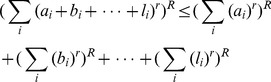
(8)where 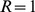, if 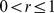 and 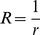, if 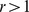.

As an extension of discrete Jensen’s inequality, the following inequality has been derived in [22].

**Lemma 3** [22] *Let* 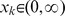 *, for* 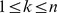 *, and* 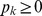 *such that* 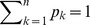 *. Then.*

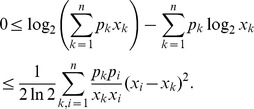
(9)


## Results and Discussion

In this section, we present our main results on implicit information inequalities. To begin with, we establish the bounds for Rényi entropy in terms of Shannon entropy.

**Theorem 4** *Let 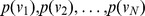 be the probability values on the vertices of a graph G. Then the Rényi entropy can be bounded by the Shannon entropy as follows*:

When 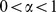,
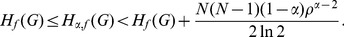
(10)


*When* 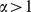,
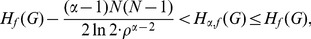
(11)


*where* 
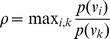
.

**Proof:** It is well known [35] that the Rényi entropy satisfies the following relation with the Shannon entropy.
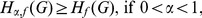
(12)and



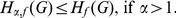
(13)To prove the bound for 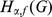, let 
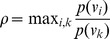
. Consider, the inequality (9) from Lemma 3 with 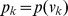 and 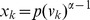. We get,
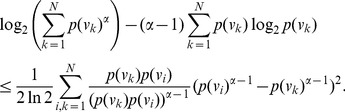
(14)


Now we prove the theorem by considering intervals for 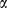.

**Case 1:** When 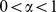.

Dividing by 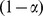 on either side of the expression (14), we get.
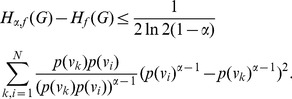
(15)


Applying inequality (6) from Lemma 1 to the term 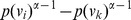 with 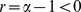 in the above sum, we obtain.
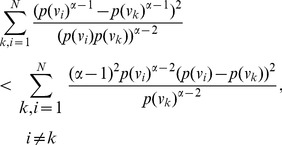
(16)

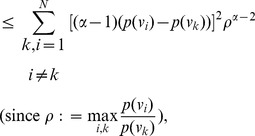
(17)

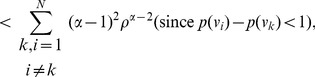
(18)

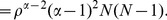
(19)


Now expression (15) becomes.
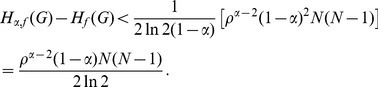
(20)Thus,



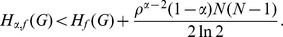
is the desired upper bound in (10).

**Case 2:** When 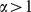.

In this case dividing by 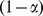 on either side of the expression (15), we get,
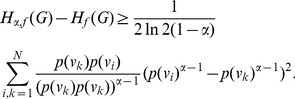
(21)


When 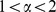, we have 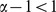. Therefore by applying inequality (7) to the term 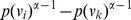 with 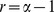 in the above sum we get,
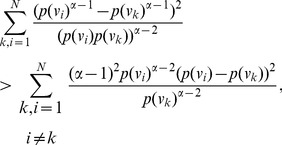
(22)

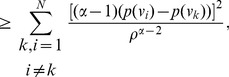
(23)

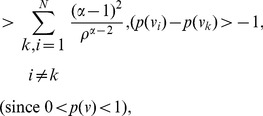
(24)

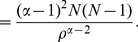
(25)


Note that when 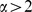, by applying inequality (6), as before, to the term 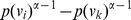 with 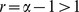 and by simplifying we get the same expression as above. When 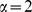, by direct simplification we get a similar expression. Hence we conclude that the expression (25) holds, in general for 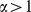.

Therefore by substituting inequality (25) in (21), we get.
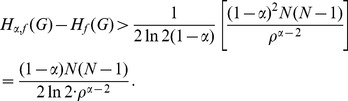
(26)Thus,



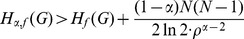
.is the desired lower bound in (11).

**Corollary 5** *In addition, suppose* 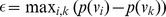 *, then.*

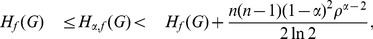
(27)

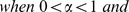


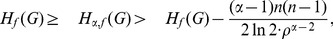
(28)when 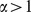.

**Remark 6** Observe that Theorem 4, in general, holds for any arbitrary probability distribution with non-zero probability values. The following theorem illustrates this fact with the help of a probability distribution obtained by partitioning a graph object.

**Theorem 7** *Let* 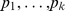 *be the probabilities of the partitions obtained using an equivalence relation* 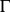 *as stated before. Then.*

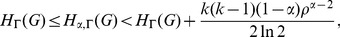
(29)
*When* 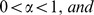


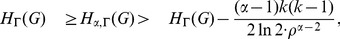
(30)when 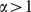. Here 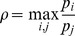.

**Proof:** By proceeding similarly to Theorem 4, we get the desired result.

In the next theorem, we establish bounds between like-entropy measures, by considering the two different probability distributions.

**Theorem 8** *Suppose* 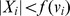 *, for* 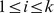 *, then.*

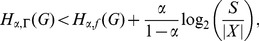
(31)

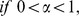


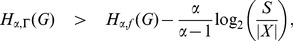
(32)if 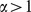. Here 
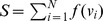
.

**Proof:** Let 
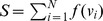
 and thus 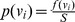. Now, given 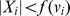, for 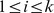 we have,
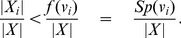
(33)


By raising either side of the expression to the power 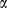, we get.
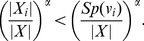
(34)


Applying summation over *i* from 1 to *k* on either side we get,
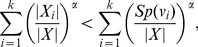
(35)

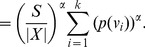
(36)

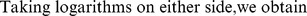


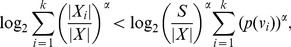
(37)

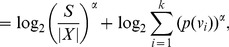
(38)

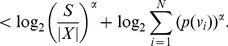
(39)


Now we distinguish two cases, depending on 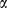 as follows:

**Case 1:** When 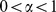, dividing by 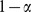 on either side of equation (39), we get.
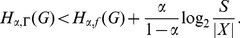
(40)


**Case 2:** When 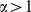, dividing by 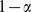 on either side of equation (39), we get.
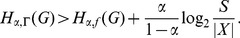
(41)


Expressions (40) and (41) are the desired inequalities.

**Remark 9** A similar relation by considering 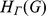 and 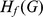 has been derived in [25].

We focus our attention to the Rényi entropy measure defined using information functionals (given by equation (5)) and present various bounds when two different functionals and their probability distributions satisfy certain initial conditions. A similar study has been performed in [17], [23] by using Shannon’s entropy only.

Let 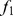 and 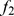 be two information functionals defined on 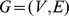. Let 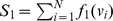 and 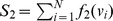. Let 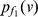 and 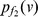 denote the probabilities of 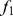 and 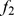, respectively, on a vertex 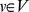. Let 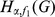 and 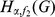 denote the Rényi entropy based on the functionals 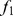 and 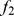 respectively.

**Theorem 10** *Suppose* 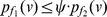 *,* 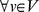 *and* 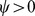 *a constant, then.*

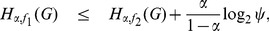
(42)

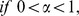


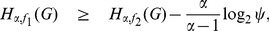
(43)if 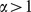.

**Proof:** Given.
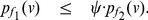
(44)

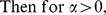


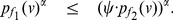
(45)


Applying summation over the vertices of *G*, we get.
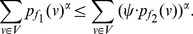
(46)

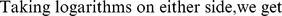


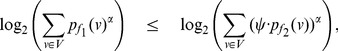
(47)

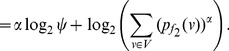
(48)


**Case 1:** When 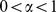. Dividing either side of the equation by 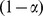 yields the desired expression (42).

**Case 2:** When 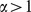. In this case, dividing either side of the equation by 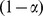 yields the expression (43) as desired.

**Corollary 11** *Suppose* 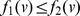 *,* 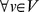 *, then.*

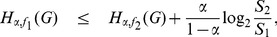
(49)

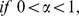


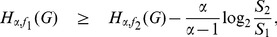
(50)if 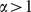.

**Proof:** By assumption, we have 
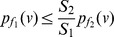
. Therefore, the corollary follows by letting 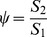 in the above theorem.

The next theorem can be used to study how a minor perturbation in the probability distribution of the system can affect the corresponding value of Rényi entropy measure. The amount of deviation can then be estimated as follows.

**Theorem 12** Suppose 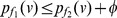, 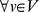 and 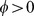 a constant, then.
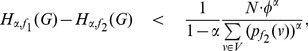
(51)

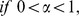


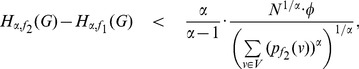
(52)if 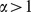.

**Proof:** Suppose 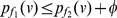, 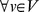. Then.
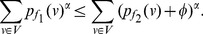
(53)


**Case 1:** When 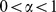.

By applying Lemma 2 with 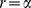, 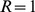, 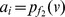 and 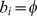, in expression (53) we get,
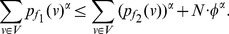
(54)

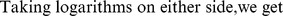


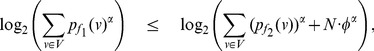
(55)

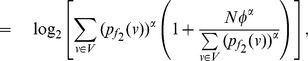
(56)

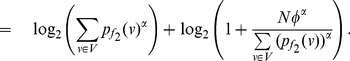
(57)


It is well known that 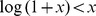, for 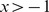. Using this relation for the second term in the above expression, we get.
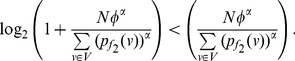
(58)


Thus, (57) can be expressed asp.
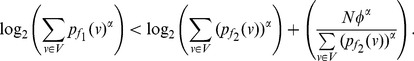
(59)


Dividing by 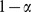, yields the desired expression (51).

**Case 2:** When 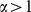.

By applying Lemma 2 with 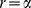, 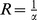, 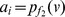 and 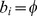 to expression (53) we get,
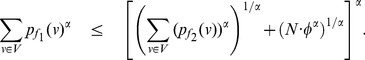
(60)

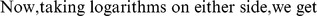


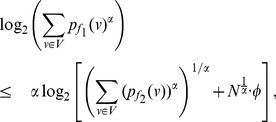
(61)

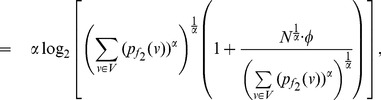
(62)

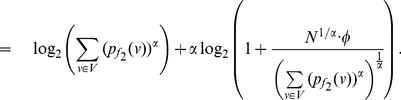
(63)


Using the relation 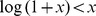 (for 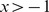), in the above expression, we get.
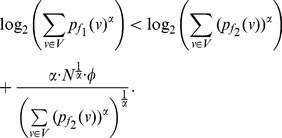
(64)


Dividing by 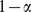, yields the desired expression (52).

**Theorem 13** *Let* 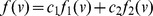 *,* 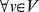 *. Then,*

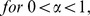


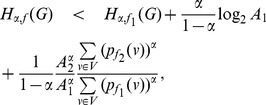
(65)

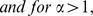


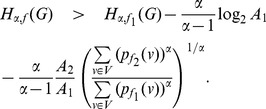
(66)


*Here,* 
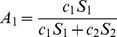
 *and* 
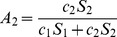
.

**Proof:** Consider, 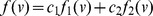, 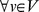. Now let 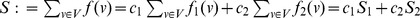. Next consider,
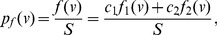
(67)

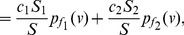
(68)

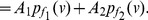
(69)


Then for 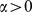, we have.
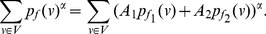
(70)


Case 1: 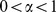.

Applying Lemma 2 with 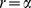, 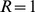, 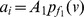 and 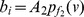 in expression (70), we get.
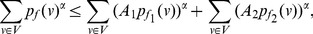
(71)

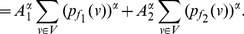
(72)


Taking logarithms on either side, we get.
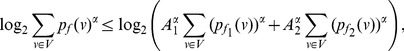
(73)

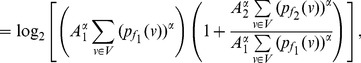
(74)

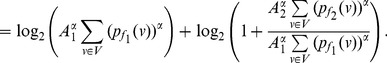
(75)


Using the relation 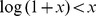 (for 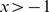), in the above expression, we get.
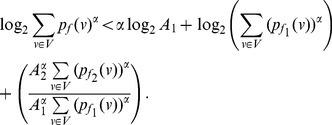
(76)


Dividing by 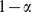, yields the desired expression (65).

**Case 2: 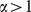.**

Applying Lemma 2 with 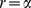, 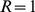, 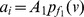 and 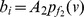 in expression (70), yields.
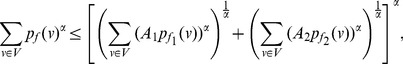
(77)

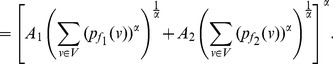
(78)


Taking logarithms on either side, we get.
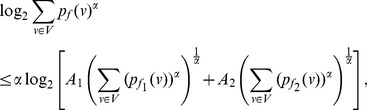
(79)

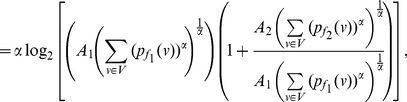
(80)

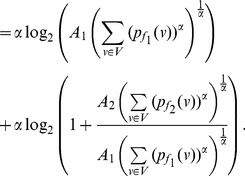
(81)


Using the relation 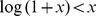 (for 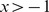), in the above expression, we get.
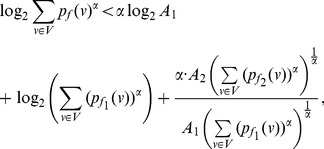
(82)


Dividing by 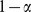, yields the desired expression (66).

**Corollary 14** *Let 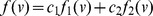, 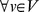.*If 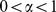, then
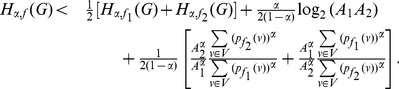
(83)If 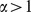, then
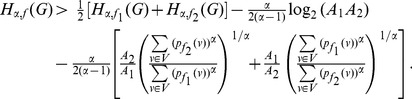
(84)Here, 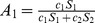 and 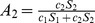.

**Proof:** The proof follows similarly to Theorem 13. In case of 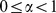, the equation (73) can be expressed as follows:
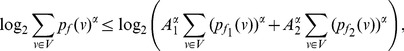
(85)

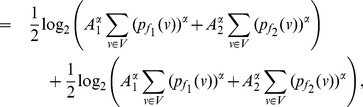
(86)

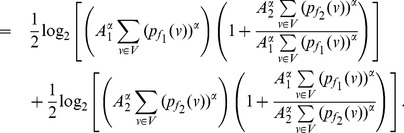
(87)


Finally by proceeding as before and by simplifying each of the terms in the above equation, we get the desired expression (83).

Similarly as in the case of 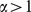, the expression (79) can be expressed by,
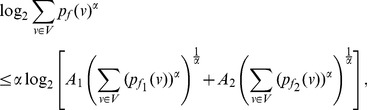
(88)

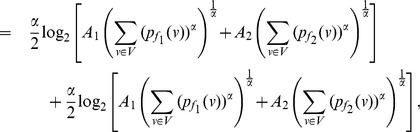
(89)

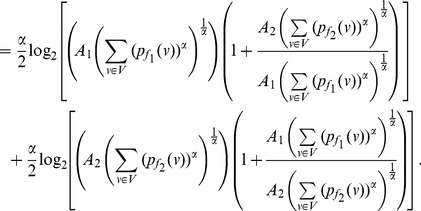
(90)


Upon simplification of the above equation, we get the desired expression (84).

### Applications to chemical graphs

In this section, we consider various classes of chemical graphs and illustrate the results from the previous section. To this purpose, we consider a specific example of the equivalence relation 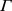 on *G* and an information functional 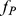. In order to define concrete graph entropies, we need to specify graph invariants and information functionals to determine a probability distribution.

For the graph invariant we use the automorphism group of a graph. We use this invariant due to their extensive investigations available in the literature; for example see [27]. Note that there are various other invariants such as distance, degrees and paths that could be used. Observe that each graph belongs to an automorphism group, where an automorphism is a permutation of the vertices such that the adjacency relation of the graph is preserved. An automorphism group divides the vertex set into orbits where a vertex orbit is a collection of topologically equivalent vertices [27].

**Definition 5** *Let 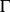 be an automorphism (equivalence relation) that partitions the vertex set V of G into vertex orbits. Let 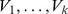 be the k orbits of V such that* 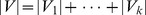.

As to the information functional, we reproduce the definitions of two information functionals based on metrical properties of graphs [18], [29], [30].

Let 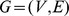 be a simple, undirected graph on *n* vertices and let 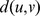 denote the distance between two vertices *u* and *v*, and let 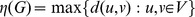. Let 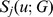 denote the *j*-sphere of a vertex *u* defined as 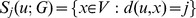.

**Definition 6** *Parameterized linear information functional using j-spheres* [*18]*, [*29*] *:*

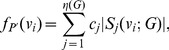
(91)where 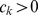 for 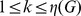.

**Definition 7** *Parameterized exponential information functional using j-spheres* [*18]*, [*29*] *:*

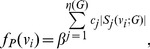
(92)where 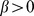 and 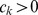 for 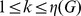.

**Remark 15** *The setting* 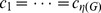 *is trivial as* 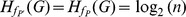 *. But anyway, for all combinations of* 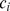 *the resulting measures are well defined.*

Note that the constants 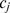 in the above expressions contribute to the weight of the *j*-spheres, see [36]. If 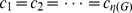, see Remark 15. When the 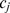 are all distinct, the vertices belonging to different *j*-spheres are weighted differently while the vertices belonging to the same *j*-sphere are considered to have same weight. Interestingly, the choice of constants 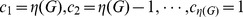 has been proven useful for solving problems in chemical graph analysis [36]. By doing so, the emphasis of a particular vertex is mainly given by its nearest neighbors and that the contribution of vertices at farthest distance is low. For more examples, we refer to [29], [37].

For the rest of the article, we consider two graph classes namely the stars and the path graphs to show the application of results from previous section. In addition, we also present the behavior of certain information functionals for any general connected graphs. A similar analysis on the relation between Shannon entropy measure (only) has been performed in [17], [25].

### Stars

A Star 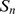 is a tree on *n* vertices where there is exactly one vertex of degree 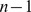 and 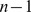 vertices of degree 1, see [38]. The unique vertex of degree 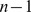, denoted by *u*, is also referred to as *central vertex*. Star graphs have been of considerable interest, since they represent trees with smallest possible diameter among all trees on *n* vertices. Let 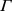 be an automorphism defined on 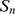 such that 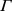 partitions 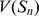 into two orbits, 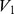 and 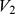, where 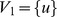 and 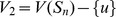.

**Theorem 16** *If 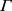 is the automorphism, as defined above, on 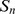. Then.*



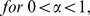


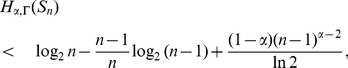
(93)

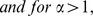


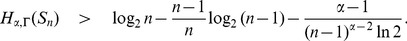
(94)


**Proof:** Let 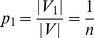 and 
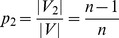
. So, 
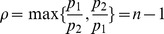
. Now, we have.
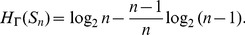
(95)

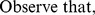


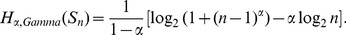
(96)


Now by Theorem 7, we have.
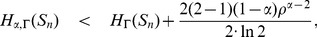
(97)

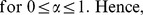


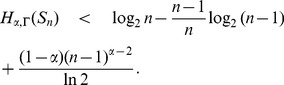
(98)


Similarly, for 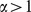, we have by Theorem 7,
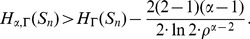
(99)

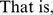


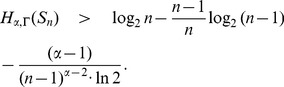
(100)


Hence, the theorem follows.

**Theorem 17** *Let* 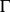 *be an automorphism on* 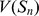 *and let f be any information functional defined on* 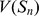 *such that* 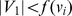 *and* 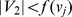 *for some i and j,* 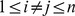 *. Then, for* 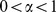 *,*

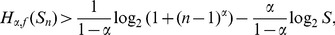
(101)

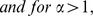


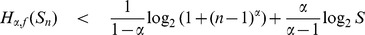
(102)Here 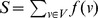.

**Proof:** Follows by using equation (96) in Theorem 8.

**Remark 18** Observe that since 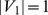 and 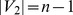, there exists functionals satisfying the conditions of the theorem. For instance, if 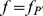 defined by equation (91) then.
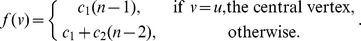



When 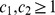, the conditions of the theorem are satisfied. That is, 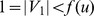 and 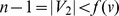, for some 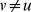. Note we obtain a family of functionals (depending on 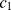 and 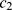) satisfying the conditions of the theorem. Also, we have 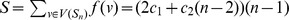. By substituting the value of *S* in expressions (101) and (102), we get the bounds for 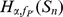.

**Remark 19** Another interesting graph class possessing the same automorphism group as the stars is the class of wheel graphs. A wheel 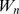 is a graph obtained by joining a new vertex *v* to every vertex of an 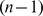-cycle 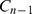. That is, 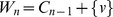. While studying the inequalities for this class of graph, we derived similar expressions as of theorems 16 and 17. Hence, we conclude that the theorems 16 and 17 also holds for the wheel 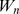.

### Paths

A path graph, denoted by 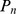, are the only trees with maximum diameter among all the trees on *n* vertices. This class of graph has received considerable attention in chemistry when studying the hydrogen-depleted hydrocarbon molecules. Let 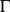 be an automorphism defined on 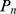, where 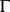 partitions the vertices of 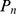 into 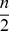 orbits (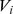) of size 2, when *n* is even, and 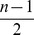 orbits of size 2 and one orbit of size 1, when *n* is odd.

In the following theorem, we consider 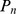, when *n* is even.

**Theorem 20** *Let n be an even integer and f be any information functional such that* 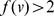 *for at least* 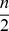 *vertices of* 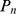 *and let* 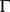 *be as defined above. Then.*

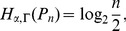
(103)

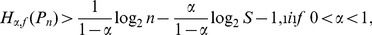
(104)

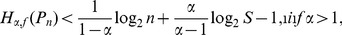
(105)where 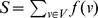.

**Proof:** *Since n is even,* 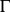 *partitions* 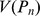 *into* 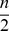 *orbits of size 2. That is, for* 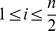 *,* 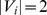 *. Therefore,* 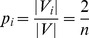 *, for* 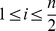 *.* 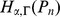 *is derived as follows*

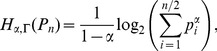
(106)

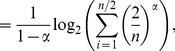
(107)

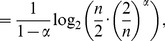
(108)

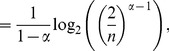
(109)

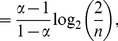
(110)

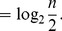
(111)


Next, by using this value of 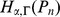 and 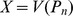 in Theorem 8, we get the desired expression for 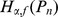.

When we consider 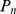, *n* being odd, evaluating 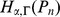 is not immediate. Hence we invoke Theorem 7 and obtain the following result.

**Theorem 21** *Let n be an odd integer and let* 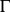 *be defined as before. Then.*

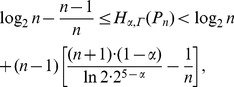
(112)

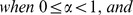


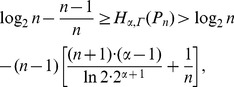
(113)when 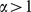. Further if f is an information functional such that 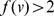 for at least 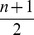 vertices of 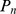, then
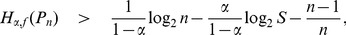
(114)

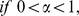


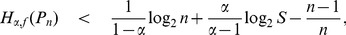
(115)if 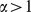. Here 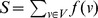.

**Proof:** Since *n* is odd, 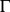 partitions 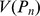 into 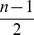 orbits of size 2 and one orbit of size 1. That is, 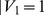, and for 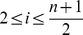, 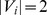. Therefore, 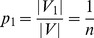, and for 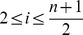, 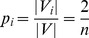.

First we compute the Shannon entropy 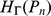 as follows.
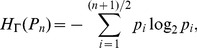
(116)

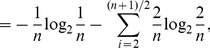
(117)

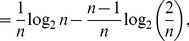
(118)

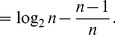
(119)


By using this value of 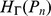 along with 
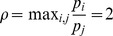
 and 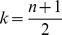 in Theorem 7, we get the desired bounds for 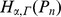.

Next we evaluate the bounds for 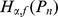.

First let 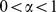. Consider expression (31) from Theorem 8. That is,
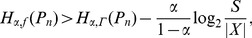
(120)

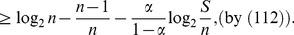
(121)


Upon simplification of the above expression, we get the desired bound (114).

In the case of 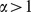, by proceeding similarly using expression (32) from Theorem 8, we yield the other bound (115).

**Remark 22** *Observe that, the computation of the Rényi entropy even with the classical partition-based distributions is not immediate for odd paths when compared to even paths. Hence, getting a closed form expression for general connected graphs is equally difficult.*

### Connected graphs

In this section, we consider any general connected graph *G* on *n* vertices and the functionals 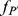 and 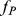 given by equations (91) and (92) respectively. In the next two theorems, we present the explicit bounds for the Rényi entropy 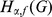, when we choose the two information functionals in particular.

**Theorem 23** *Let* 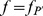 *given by* *equation (91* *). Let* 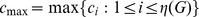 *and* 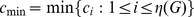 *where* 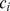 *is defined in* 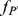 *. Then the value of* 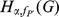 *lies within the following bounds.*

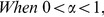
(122)

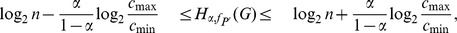


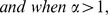


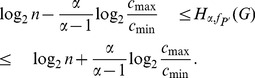
(123)


**Proof:** Given 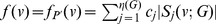 with 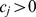 for 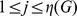. Let 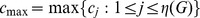 and 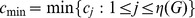. We have,
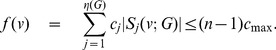
(124)

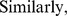


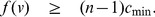
(125)


Therefore, combining the Equations (124) and (125) and by adding over all the vertices of *G*, we get.
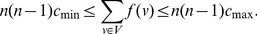
(126)

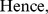


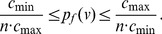
(127)

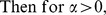


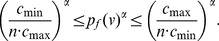
(128)


Applying summation over all the vertices of *G*, we obtain.
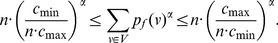
(129)

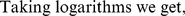


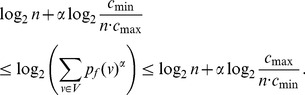
(130)


Dividing the expression (130) by 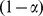, and simplifying we get the desired expressions given by (122) and (123) depending on the value of 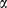.

**Figure 1:**
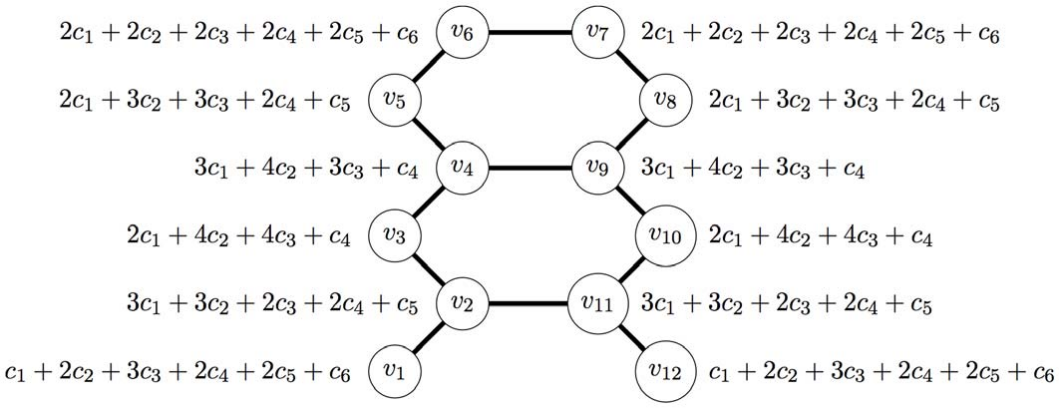
A Graph *G* along with the value of 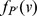.

Let us illustrate the above theorem by the following example. Let 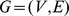 be the graph on 12 vertices as shown in Figure 1. The corresponding value of the information functional 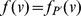 is also depicted in Figure 1. Here, 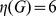. Also, 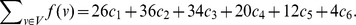


It is known that 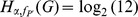 (see Remark 15) if 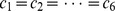. Equivalently, by using Theorem 23, we arrive at the same value, since 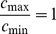 and that 
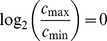
. Observe that, the upper and lower bounds of 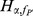 coincides with this choice of constants.

Let us illustrate a nontrivial case by setting the constants for 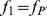 as follows [18], [29]:
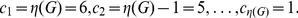
(131)


Hence the Rényi entropy then becomes.
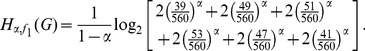
(132)


Finally, we obtain.
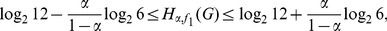
(133)if 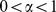, and
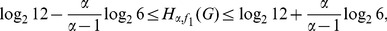
(134)if 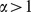.

**Theorem 24** *Let 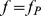 given by* *equation* (92). *Let 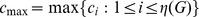 and 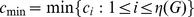 where 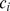 is as defined in 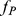. Then the value of 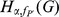 can be bounded as follows.*



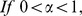
.
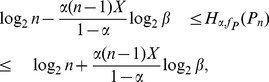
(135)




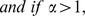


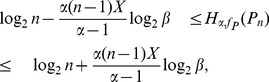
(136)where 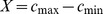.

**Proof:** Given 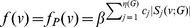 with 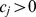 for 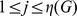. Let 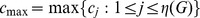 and 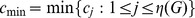. We have,
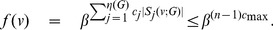
(137)

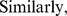


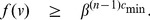
(138)


Therefore, combining the Equations (137) and (138) and adding over all the vertices of *G*, we get.
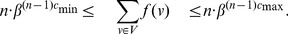
(139)

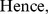


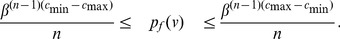
(140)


Let 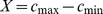. Now, by raising 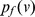 to the power 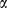 and adding over all the vertices of *G*, we have,
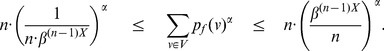
(141)

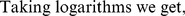


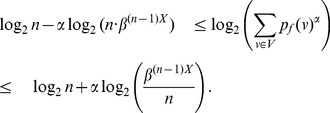
(142)

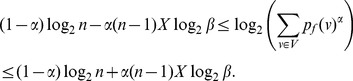
(143)


Dividing the expression (143) by 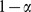, and simplifying we get the expressions (135) and (136) as desired.

### Conclusion and Summary

In this article, we have studied the problem of establishing relations between graph entropy measures. Among various entropy measures, we have considered the classical Shannon entropy and the Rényi entropy. In fact, there is only very little work when applying Rényi’s entropy to graphs [16], [39]. While this research is an extension of our earlier work [17], the results obtained here are complementing the earlier ones and of competing interest. In particular, the main contribution of this paper was to establish implicit information inequalities involving the Shannon entropy and the Rényi entropy measures when applied to networks. Also, we have presented implicit inequalities between Rényi entropy measures having two different types of probability distributions with additional assumptions. Further we have shown the application of the derived results by using various graph classes.

As mentioned earlier, investigating relations (by means of inequalities) is crucial as relations of the values of the measures have not yet been investigated extensively. To demonstrate the importance of such inequalities exemplarily, suppose 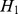 and 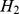 are graph entropy measures and it holds, 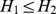 for some graph 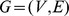. If 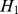 has non-polynomial complexity and 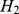 is computable in polynomial time, then 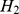 is an upper bound that may be feasible in a general sense. In terms of measures such as Körner’s entropy, relations between graph entropies could be crucial. But note that in view of the vast amount of existing measures, this is a daunting problem. Also, the meaning of the Rényi graph entropy is not yet fully understood. Hence, we believe that such relations can be useful when designing and understanding complex graph-based systems. This might be especially applicable when applying the information-theoretic network measures such as Shannon’s and Rényi’s entropy to large complex networks.

## References

[1] Bonchev D, Rouvray D (2003). Complexity in chemistry: Introduction and Fundamentals. Mathematical and Computational Chemistry 7.. New York: CRC Press.

[2] Bonchev D (2009). Information theoretic measures of complexity.. In: Meyers R, editor, Encyclopedia of Complexity and System Science, Springer, volume.

[3] Anand K, Bianconi G (2009). Entropy measures for networks: Toward an information theory of complex topologies.. Physics Review E.

[4] Costa LdF, Rodrigues FA, Travieso G, Boas PRV (2007). Characterization of complex networks: A survey of measurements.. Advances in Physics.

[5] Kim J, Wilhelm T (2008). What is a complex graph?. Physica A: Statistical Mechanics and its Applications.

[6] Balaban A, Balaban T (1991). New vertex invariants and topological indices of chemical graphs based on information on distances.. Journal of Mathematical Chemistry.

[7] Bertz SH (1983). A mathematical model of complexity.. In: King R, editor, Chemical applications of topology and graph theory, Elsevier, Amsterdam..

[8] Basak SC, Magnuson VR, Niemi GJ, Regal RR (1988). Determining structural similarity of chemicals using graph-theoretic indices.. Discrete Applied Mathematics.

[9] Bonchev D, Rouvray D (2005). Complexity in chemistry, biology, and ecology. Mathematical and Computational Chemistry. New York: Springer, xx+344 pp. doi:10.1007/b136300.. URL.

[10] Claussen JC (2007). Offdiagonal complexity: A computationally quick complexity measure for graphs and networks.. Physica A: Statistical Mechanics and its Applications.

[11] Körner J (1973). Coding of an information source having ambiguous alphabet and the entropy of graphs.. Trans 6th Prague Conference on Information Theory.

[12] Butts C (2001). The complexity of social networks: Theoretical and empirical findings.. Social Networks.

[13] Bonchev D (1983). Information Theoretic Indices for Characterization of Chemical Structures.. Research Studies Press, Chichester.

[14] Rényi P (1961). On measures of information and entropy.. In: Proceedings of the 4th Berkeley Symposium on Mathematics, Statistics and Probability. Berkeley, CA: University of California Press, volume 1, 547–561..

[15] Daròczy Z, Jarai A (1979). On the measurable solutions of functional equation arising in information theory.. Acta Math Acad Sci Hungar.

[16] Dehmer M, Mowshowitz A (2011). Generalized graph entropies.. Complexity.

[17] Dehmer M, Sivakumar L (2012). Recent Developments in Quantitative Graph Theory: Information Inequalities for Networks.. PLoS ONE.

[18] Dehmer M, Mowshowitz A (2011). A history of graph entropy measures.. Information Sciences.

[19] Cover TM, Thomas JA (2006). Elements of Information Theory.Wiley Series in Telecommunications and Signal Processing.. Wiley & Sons.

[20] Dembo A, Cover T, Thomas J (1991). Information theoretic inequalities.. IEEE Tranactions on Information Theory.

[21] Yeung RW (1997). A framework for linear information inequalities.. IEEE Transactions on Information Theory.

[22] Dragomir SS, Goh CJ (1997). Some bounds on entropy measures in information theory.. Applied Mathematics Letters.

[23] Dehmer M, Mowshowitz A (2010). Inequalities for entropy-based measures of network information content.. Applied Mathematics and Computation.

[24] Bonchev D, Trinajstić N (1977). Information theory, distance matrix, and molecular branching.. The Journal of Chemical Physics.

[25] Dehmer M, Mowshowitz A, Emmert-Streib F (2011). Connections between classical and parametric network entropies.. PLoS ONE.

[26] Dehmer M, Borgert S, Emmert-Streib F (2008). Entropy bounds for hierarchicalmolecular networks.. PLoS ONE.

[27] Mowshowitz A (1968). Entropy and the complexity of graphs: I. an index of the relative complexity of a graph.. Bulletin of Mathematical Biophysics.

[28] Dehmer M (2008). Information-theoretic concepts for the analysis of complex networks.. Applied Artificial Intelligence: An International Journal.

[29] Dehmer M (2008). Information processing in complex networks: Graph entropy and information functionals.. Applied Mathematics and Computation.

[30] Skorobogatov VA, Dobrynin AA (1988). Metrical analysis of graphs.. MATCH Commun Math Comp Chem.

[31] Shannon CE (1948). A mathematical theory of communication.. Bell System Technical Journal 27: 379–423 and 623–656.

[32] Hardy GH, Littlewood JE, Pólya G (1988). Inequalities. Cambridge Mathematical Library.. Cambridge University Press; 2 edition.

[33] Shannon C, Weaver W (1997). The Mathematical Theory of Communication.. University of Illinois Press, Urbana, IL, USA.

[34] Rashevsky N (1955). Life, information theory and topology.. Bulletin of Mathematical Biophysics.

[35] Arndt C (2004). Information Measures: Information and its Description in Science and Engineering (Signals and Communication Technology).. Springer.

[36] Dehmer M, Emmert-Streib F (2008). Structural information content of networks: Graph entropy based on local vertex functionals.. Computational Biology and Chemistry.

[37] Dehmer M, Barbarini N, Varmuza K, Graber A (2009). A large scale analysis of informationtheoretic network complexity measures using chemical structures.. PLoS ONE.

[38] Harary F (1969). Graph Theory. Addison Wesley Publishing Company.. Reading, MA, USA.

[39] Delgado-Soler L, Toral R, Tomás MS, Rubio-Martinez J (2009). Red: A set of molecular descriptors based on ŕenyi entropy.. Journal of Chemical Modeling and Information.

